# The Mechanism and Latest Progress of m6A Methylation in the Progression of Pancreatic Cancer

**DOI:** 10.7150/ijbs.104407

**Published:** 2025-01-13

**Authors:** Ze-Hao Liu, Peng Ma, Ying He, Yue-Feng Zhang, Zuo Mou, Ting Fang, Wei Wang, Kai-Huan Yu

**Affiliations:** 1 Department of Hepatobiliary Surgery, Renmin Hospital of Wuhan University, Wuhan, 430060, China.; 2 Department of Hepatobiliary Surgery, East Hospital, Renmin Hospital of Wuhan University, Wuhan, 430060, China.; 3 Department of Stomatology, Renmin Hospital of Wuhan University, Wuhan, 430060, China.; 4 Department of Hepatobiliary Surgery, East Hospital, Renmin Hospital of Wuhan University, Wuhan, 430060, China.; 5 Department of Hepatobiliary Surgery, Renmin Hospital of Wuhan University, Wuhan, 430060, China.; 6 Department of Oncology, Renmin Hospital of Wuhan University, Wuhan, 430060, China.; 7 Department of Hepatobiliary Surgery, East Hospital, Renmin Hospital of Wuhan University, Wuhan, 430060, China.; 8 Department of Hepatobiliary Surgery, East Hospital, Renmin Hospital of Wuhan University, Wuhan, 430060, China.

**Keywords:** Pancreatic cancer, RNA Methylation, m6A, Immune Evasion, RNA, Untranslated

## Abstract

Pancreatic cancer (PC), known as the "king of cancers," is characterized by an exceptionally low five-year survival rate, posing a formidable challenge to global public health. N6-methyladenosine (m6A) methylation is prevalent across various stages of eukaryotic RNA expression, including splicing, maturation, stability, translation, and localization, and represents a pivotal mechanism of epigenetic regulation. m6A methylation influences tumor initiation and progression by modulating post-transcriptional processes, playing a critical role in sustaining cancer cell stemness, promoting cell proliferation, and mediating drug resistance. Extensive research underscores the substantial contribution of m6A modifications to PC development. However, the multiplicity of m6A regulators and their intricate mechanisms of action complicate the landscape. This review aims to deepen the understanding of m6A's role in PC by delineating its involvement in four key areas of tumorigenesis: the hypoxic tumor microenvironment, metabolic reprogramming, immune microenvironment, and resistance mechanisms. Additionally, the review addresses the emerging frontier of m6A interactions with non-coding RNAs (ncRNAs), offering insights into the potential therapeutic and prognostic applications of m6A in the treatment and prognosis prediction of PC.

## 1. Introduction

Pancreatic cancer (PC), known as the "king of cancers," remains a leading cause of cancer-related mortality. According to the latest data from the CA: A Cancer Journal for Clinicians, PC was the sixth leading cause of cancer deaths globally in 2022, accounting for 4.8% of all cancer fatalities worldwide. Mortality rates for PC have remained relatively stable across many countries in recent decades^1^. Projections indicate that by 2025, PC will become the third leading cause of cancer-related deaths in Europe, and by 2030, it is expected to rank second in the United States. As a highly aggressive malignancy with a poor prognosis, PC represents a significant public health burden^2^-^4^. The majority of PC cases present as pancreatic ductal adenocarcinoma (PDAC), which is marked by rapid growth, aggressive invasiveness, a high degree of malignancy, and typically lacks discernible early symptoms. This results in delayed diagnosis, often until the disease has reached an advanced stage or metastasis^5^, ^6^. Gemcitabine (GEM)-based combination chemotherapy remains the primary treatment for advanced PDAC; however, the emergence of drug resistance has severely hindered the efficacy of this regimen in recent years^7^, ^8^. Consequently, a deeper understanding of the molecular mechanisms driving PC initiation, progression, and drug resistance, along with insights into the role of the tumor microenvironment, is urgently needed.

N6-methyladenosine (m6A) modification, first identified in the 1970s, has garnered significant attention as the most prevalent modification of messenger RNA (mRNA) and long non-coding RNA (lncRNA) in eukaryotes^9^. m6A modification involves the methylation of adenosine at the N6 position, a reversible modification regulated by a dynamic interplay of methyltransferases ("writers"), demethylases ("erasers"), and RNA-binding proteins ("readers") that recognize and interpret the methylation marks^10^. m6A methylation is widespread in mammalian mRNA and has been identified in various RNA species, including transfer RNA (tRNA), ribosomal RNA (rRNA), circular RNA (circRNA), microRNA (miRNA), and lncRNA^11^. This modification plays a pivotal role in regulating gene expression in tumor cells by influencing RNA processes such as splicing, maturation, stability, translation, and localization, thereby controlling cancer progression^12^. Several studies have highlighted the essential role of m6A in cancer, particularly its influence on the proliferation, development, and metastasis of PC^13^-^15^. In a recent review, Wang *et al.* explored the epigenetic regulation of m6A regulators themselves, detailing post-translational modifications such as ubiquitination, SUMOylation, acetylation, methylation, phosphorylation, O-GlcNAcylation, ISGylation, and lactylation, all of which impact cancer progression^16^. However, comprehensive and up-to-date reviews on the mechanisms of m6A modification in PC remain scarce. This review aims to consolidate recent advances in m6A research in the context of PC, focusing on four key aspects: the hypoxic tumor microenvironment, metabolic reprogramming, immune microenvironment, and drug resistance mechanisms. Additionally, the review addresses emerging research on the interaction between non-coding RNAs (ncRNAs) and m6A methylation. By providing an in-depth understanding of m6A modification in PC, this review aims to contribute to the development of improved non-surgical treatment strategies for patients with PC, ultimately improving their clinical outcomes.

## 2. m6A modifiers

m6A writers, also referred to as methyltransferases, are enzymes that utilize S-adenosyl methionine (SAM) as a cofactor to catalyze N6-methylation by transferring a methyl group from SAM to the adenosine substrate^17^. These writers typically function as multifunctional complexes composed of several key components, including methyltransferase-like 3 (METTL3), methyltransferase-like 5 (METTL5), methyltransferase-like 14 (METTL14), methyltransferase-like 16 (METTL16), William's tumor 1-associated protein (WTAP), RNA-binding motif protein 15/15B (RBM15/15B), vir-like m6A methyltransferase-associated protein (VIRMA/KIAA1429), phosphorylated CTD-interacting factor 1 (PCIF1), and zinc finger CCCH-type containing proteins 4/13 (ZC3H4, ZC3H13)^18^-^20^.

m6A erasers are demethylases responsible for removing m6A marks, thereby regulating the dynamic processes of m6A methylation. Notable m6A erasers include fat mass and obesity-related protein (FTO) and alpha-ketoglutarate-dependent dioxygenase alkB homolog 5 (ALKBH5). These enzymes are frequently dysregulated in cancer and play pivotal roles in malignancy development^21^, ^22^. FTO primarily removes m6A methylation from mRNA in the nucleus, while ALKBH5 acts predominantly in the cytoplasm, where it catalyzes the demethylation of m6A marks by co-binding with nuclear speckles^23^.

m6A readers are proteins that recognize m6A modifications, facilitating RNA-protein interactions and influencing regulatory pathways, such as RNA splicing, export, translation, and degradation^24^. These readers exhibit specific preferences for m6A methylation sites. Nuclear m6A readers include YTHDC1, HNRNPA2B1, HNRNPC11, and HNRNPG, while cytoplasmic m6A readers consist of YTHDF1, YTHDF2, YTHDF3, YTHDC2, as well as IGF2BP1, IGF2BP2, and IGF2BP3^11^. Recent research has identified additional m6A readers, such as the KH-type splicing regulatory protein (KHSRP), which plays an oncogenic role in PDAC progression^25^. New m6A readers have also been discovered, including fragile X mental retardation protein (FMRP), proline-rich coiled-coil 2A (PRRC2A), RNA-binding motif protein 33 (RBM33), and Eukaryotic Initiation Factor 3 (eIF3)^16^. Different m6A readers mediate diverse functions upon binding m6A-modified sites. For instance, YTHDF3 works with YTHDF1 to promote the translation of methylated RNA while also accelerating mRNA decay *via* interaction with YTHDF2. In contrast, IGF2BPs stabilize their target mRNAs in an m6A-dependent manner^26^.

**Figure 1:**
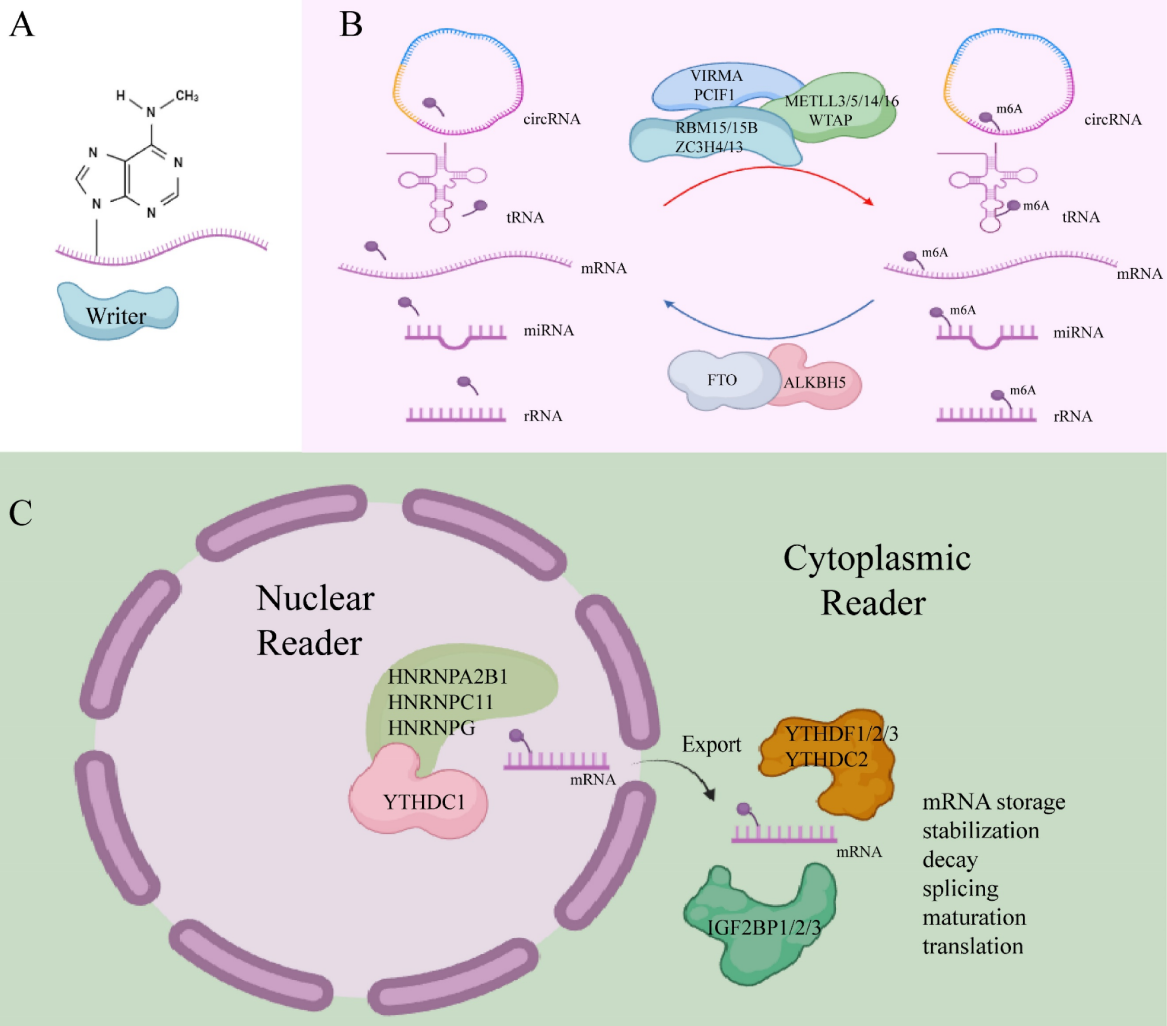
m6A writer, eraser, and reader. (Created with BioRender.com).

Together, m6A writers and erasers facilitate the reversible methylation of m6A, while m6A readers participate in the post-transcriptional regulation of RNA (Figure 1). This coordinated system represents an emerging molecular mechanism for regulating gene expression at the post-transcriptional level. Increasing evidence links m6A modification to the initiation and progression of PC (Table 1).

## 3. The pathogenesis role of m6A modulation in PC

### 3.1 Tumor hypoxic microenvironment

Hypoxia is a common feature of solid tumors, and the extent of hypoxic regions in tumor tissues is a critical factor associated with poor survival outcomes in patients with PC^27^. The m6A modification, as part of the RNA regulatory landscape, undergoes methylation changes in response to hypoxic conditions, thereby influencing tumor initiation and progression^28^. For example, in breast cancer, lncRNA KB-1980E6.3 stabilizes c-Myc mRNA under hypoxic conditions by interacting with the m6A reader IGF2BP1, thus maintaining the stemness of cancer stem cells^29^. In the context of PC, hypoxia has been shown to stimulate the expression of the m6A writer METTL3, which activates the MAPK/ERK/PD-L1 signaling pathway by modulating the mRNA stability and expression of Integrin Beta 1 (ITGB1). This process promotes the proliferation, migration, and invasion of PC cells^30^, ^31^. Additionally, hypoxic conditions significantly reduce the expression of the lncRNA GATA6-AS1 in PDAC. GATA6-AS1 disrupts the stability of SNAI1 mRNA in an m6A-dependent manner by inhibiting FTO, thus suppressing hypoxia-induced PDAC progression and epithelial-mesenchymal transition (EMT)^32^. These findings suggest that targeting the inhibition of FTO expression under hypoxic conditions may be a potential strategy to alleviate or reverse hypoxia-induced EMT in PC.

Cellular responses to hypoxia are primarily mediated by Hypoxia Inducible Factors (HIFs), a family of four subunits: HIF-1α, HIF-2α, HIF-3α, and HIF-1β. These HIF subunits form functional complexes composed of one HIF-α subunit and one HIF-1β subunit^33^, ^34^. HIFs play a significant role in PDAC progression. Specifically, HIF-2α expressed by cancer-associated fibroblasts (CAFs) within the tumor microenvironment (TME) influences tumor fibrosis, and the absence of CAF-HIF2 significantly inhibits the intratumoral recruitment of M2 macrophages and regulatory T cells (Tregs) and modestly reduced tumor fibrosis^35^. Numerous studies have demonstrated the relationship between HIFs and tumor progression, as well as their close association with m6A modifications. For example, in hepatocellular carcinoma (HCC), under hypoxic conditions, HIF-1α directly binds to the promoter region of the m6A reader YTHDF1, promoting the translation of autophagy-related genes such as ATG2A and ATG14, which drive autophagy^36^. HIF-1α also promotes metastasis in HCC through the METTL16/lnc-CSMD1-7/RBFOX2 axis^37^. In PC, methyltransferase VIRMA has been shown to target the m6A site of STRA6 mRNA, modifying it. IGF2BP2 then recognizes this m6A modification and enhances mRNA stability, leading to the activation of HIF-1α, which drives glycolysis in PDAC cells and contributes to poor prognosis^38^. Early glycolytic activation mediated by HIF-1α also promotes the migration of dendritic cells (DCs) to lymph nodes. However, stimulation by CC chemokine receptor 7 (CCR7) inhibits DC migration and affects PDAC progression by mediating m6A demethylation of lnc-Dpf3. This reduces the YTHDF2-m6A-dependent RNA degradation of lnc-Dpf3, which ultimately impacts the immune response within the TME^39^, ^40^.

In summary, m6A methylation modifications play an essential role in regulating various signaling pathways and metabolic processes in PC cells, as well as in other cells within the TME under hypoxic conditions. Targeting the modulation of m6A progression, either by inhibiting or activating specific components of the m6A machinery, may offer promising non-surgical therapeutic strategies for patients with PC.

### 3.2 Tumor metabolic reprogramming

In 2011, Hanahan proposed six hallmarks of cancer and two enabling characteristics: Sustaining proliferative signaling, Resisting cell death, Inducing angiogenesis, Evading growth suppressors, Activating invasion and metastasis, Enabling replicative immortality, alongside Reprogramming Energy Metabolism and Evading Immune Destruction^41^. Tumor metabolic reprogramming arises from mutations in oncogenes and tumor suppressor genes, with alterations in metabolite levels impacting cellular signaling, epigenetic regulation, and gene expression^42^. The regulation of m6A methylation is crucial in tumor cell metabolism, influencing growth, proliferation, and chemoresistance^43^.

PC, in particular, is characterized by a notably hypoxic microenvironment compared to other solid tumors^44^. In hypoxic PC cells, the overall mRNA m6A modification level, mediated by the eraser ALKBH5, is markedly reduced, which promotes glycolysis in an ALKBH5-dependent manner. This is mechanistically linked to a positive feedback loop involving ALKBH5, histone deacetylase type 4, and HIF1α^45^. Glutamate, in neuronal cells, enhances m6A modification of hexokinase 2 mRNA, increasing its expression *via* METTL3 upregulation, which in turn facilitates glycolysis in PDAC cells and supports perineural invasion, a hallmark of poor prognosis and decreased survival rates in PC^27^, ^46^.

During glucose deprivation, miR-5586-5p induces the overexpression of the m6A reader YTHDF3, destabilizing DICER1 antisense RNA through m6A modification and promoting glycolysis and tumor progression in PC cells^47^. Similarly, studies have shown that circular RNA Hsa_circ_0007590 targets polypyrimidine tract binding protein 1 and upregulates the m6A reader protein YTHDF2, leading to PTEN mRNA degradation and activation of the PI3K/AKT/mTOR pathway. This reprograms glucose metabolism, driving the Warburg effect and enhancing the proliferation, migration, and invasion of PDAC cells^48^. Glycogen branching enzyme 1 (GBE1), which plays a critical role in glycogen metabolism, is significantly upregulated in PC and correlates with poor patient prognosis. This is attributed to m6A modifications by methyltransferase WTAP and the reader IGF2BP3, which enhance the stability and expression of GBE1 mRNA^49^.

Advances in lipid metabolism research have also highlighted its significance in PDAC. During GEM-induced drug resistance, transforming growth factor-beta 2 (TGFB2) expression is gradually upregulated, potentially *via* METTL14-mediated m6A modification, which stabilizes TGFB2 post-transcriptionally and promotes lipid accumulation, contributing to GEM resistance^50^. The tumor suppressor SMAD4 (Mothers against decapentaplegic homolog 4) is inactivated in approximately 50%-60% of PDAC cases, disrupting the activation of the DLG1/YAP1 pathway. In this context, FTO enhances adipogenesis and lipid accumulation by stabilizing Lysine-specific demethylase 5B (KDM5B) and activating the DLG1/hippo-YAP pathway, which fosters stemness and proliferation of PDAC cells, reducing GEM sensitivity^51^. In pancreatic neuroendocrine neoplasms, upregulation of ALKBH5 plays a pivotal role in tumor growth and lipid metabolism, likely through the enhancement of FABP5 expression, which activates the mTOR signaling pathway, boosting lipid metabolism and proliferative capacity^52^.

Abnormal amino acid (AA) metabolism plays a pivotal role in promoting tumor cell proliferation and invasion. For instance, METTL14 regulates the circular RNA circSTX6 and the 144-amino acid polypeptide encoded by circSTX6 through m6A-dependent mechanisms, thereby enhancing HCC proliferation and metastasis^53^. Glutamine (Gln), an essential and the most abundant AA in the blood, is integral to numerous biological processes that support cancer cell growth and proliferation, making it a critical component of cancer metabolism^54^. The rapid proliferation of PDAC relies on an atypical Gln utilization pathway; inhibition of this altered metabolism effectively suppresses PDAC growth, migration, and invasion^55^. In PC, SMAD nuclear-interacting protein 1 (SNIP1) recognizes and binds to m6A-modified lncRNA BCAN-AS1, preventing the ubiquitination and degradation of c-Myc, a process mediated by S-phase kinase-associated protein 2 (SKP2)^56^. Oncogenic levels of c-Myc, however, can drive transcriptional programs that promote glutaminolysis, creating cellular dependence on Gln as a bioenergetic substrate^57^. Reprogramming of Gln metabolism is a hallmark of cancer, with dysregulation of glutaminase (GLS) and glutamine synthetase (GS) identified as key events in tumor metabolic rewiring^58^. While there is limited research on the role of m6A in Gln metabolism in PC, emerging studies in other cancers have begun to elucidate this connection. For example, the m6A reader YTHDF1 promotes GLS expression in colon cancer, contributing to cisplatin resistance, while IGF2BP3 enhances glutamine and glutamic acid metabolism in cervical cancer cells by upregulating GLS expression, thereby facilitating immune evasion^59^, ^60^. Although the metabolic reprogramming of Gln is a characteristic of abnormal AA metabolism, research linking m6A modifications to this process in PC remains scarce. Drawing from studies in other malignancies could provide new avenues for targeted therapeutic strategies.

Metabolic reprogramming serves as a fundamental driver of tumor proliferation, migration, and invasion, with m6A methylation modifications playing a pivotal role in modulating metabolic pathways such as glucose, lipid, and AA metabolism. Targeting m6A regulation as a therapeutic strategy holds potential for benefiting patients with PC by prolonging survival and improving quality of life.

### 3.3 Tumor immune microenvironment

Studies proposed the association between the m6Ascore and poor overall survival as well as increased tumor recurrence in PDAC and other solid tumors, including colorectal and breast cancer. Higher m6Ascores were observed in basal-like (squamous) PDAC compared to classical PDAC. Tumors with elevated m6Ascores are characterized by diminished immune infiltration and T-cell exhaustion^61^. PC is generally regarded as an immune-impaired malignancy, where the development and activation of immune cells are heavily reliant on precise epigenetic regulation^62^. m6A mRNA methylation is an emerging post-transcriptional regulatory mechanism influencing gene expression, playing a significant role in the modulation of various immune cell functions^63^.

Macrophage polarization serves as a key indicator of the immune status within tumors and is linked to poor prognosis in patients with PDAC. In research conducted by Wang *et al.* from our team, inhibition of the m6A writer RBM15 was shown to increase macrophage infiltration, promoting phagocytosis of PC cells by macrophages, thereby establishing RBM15 as an independent prognostic factor for PC^63^. The lncRNA-PACERR, which binds to IGF2BP2, enhances the stability of KLF12 and c-Myc in the cytoplasm of tumor-associated macrophages (TAMs) in an m6A-dependent manner. PACERR, by interacting with miR-671-3p, activates the KLF12/p-AKT/c-Myc signaling pathway, increases the number of M2-polarized macrophages, and promotes the proliferation, invasion, and migration of tumor cells^64^. The m6A reader YTHDF2 inhibits MAPK and NF-κB signaling pathways by downregulating MAP2K4, MAP4K4, STAT1, and PPAR-γ expression, preventing macrophage polarization and secretion of pro-inflammatory cytokines^65^. m6A also participates in the M1 polarization of TAMs, with the m6A methyltransferase METTL3 being selectively upregulated during M1 polarization. METTL3 mediates the methylation of STAT1 mRNA, significantly enhancing its stability and expression, thereby driving M1 polarization of macrophages^66^. Depletion of METTL3 in TAMs enhances both M1 and M2-like TAMs, as well as Treg infiltration into tumors, remodeling the tumor microenvironment and promoting tumor growth and metastasis^67^.

Dong *et al.* developed an m6A scoring system, discovering a significant correlation between the m6Ascore and various immune cell types, including CD4+ T cells, Tregs, type 1 T helper cells (Th1), γδ T cells, natural killer T cells (NKT), activated DCs, CD56+ NK cells, and NK cells. Notably, high expression of RBM15 showed a positive correlation with lymphocyte counts, and silencing the RBM15 gene inhibited PC cell proliferation, migration, and metastasis^68^. In METTL3-deficient naive T cells, the m6A modification of suppressor of cytokine signaling (SOCS) family gene mRNAs (such as SOCS1, SOCS3, and CISH) was diminished, leading to slower mRNA decay compared to wild-type naive T cells. This resulted in elevated mRNA and protein levels, inhibition of IL-7-mediated STAT5 activation, and disruption of naive T cell homeostasis, proliferation, and differentiation^69^. The m6A reader YTHDF1 in DCs enhances antigen degradation, limiting the presentation of tumor neoantigens to T cells and inhibiting T cell-mediated immune responses^70^.

Neutrophils, the most abundant immune cells in human blood circulation, are essential in defending against microbial infections and play a significant role in regulating both innate and adaptive immunity. They interact with a wide range of immune cells, including macrophages, mesenchymal stem cells, DCs, NK cells, and B and T cells, forming complex bidirectional relationships within tissues^71^. In recent years, neutrophils have attracted increased attention due to their cancer-promoting roles. They contribute to cancer progression through mechanisms such as DNA damage induction, angiogenesis promotion, and immune suppression^72^. m6A methylation plays a critical role in neutrophil functions, including migration, activation, and the formation of neutrophil extracellular traps (NETs). For example, the m6A eraser ALKBH5 enhances neutrophil migration by increasing the expression of CXCR2 and NLRP12 while decreasing the expression of PTGER4, TNC, and WNK1^73^. Upon reaching the tissues, inflammatory stimuli activate neutrophils, where METTL3-mediated m6A methylation boosts TLR4 expression, regulating neutrophil activation^74^. Activated neutrophils can release NETs during the process of NETosis^75^. These NETs mediate proteolytic remodeling of laminin, which subsequently enhances cancer cell proliferation and metastasis^76^. Additionally, upregulation of IGF2BP3 in neutrophils enhances the expression of MIB1, an E3 ubiquitin-protein ligase that promotes FTO degradation *via* the ubiquitin-proteasome pathway. This results in increased m6A-mediated NET formation^77^. Thus, m6A modification plays a pivotal role in neutrophil migration, activation, and NET formation, which subsequently influences tumor initiation and progression.

Moreover, m6A modification influences immune evasion mechanisms in tumor cells by regulating immune-related signaling pathways. METTL3, for instance, positively regulates the expression of lncRNA MALAT1 in PC cells, which enhances PD-L1 expression and modulates tumor immune surveillance^78^. Additionally, METTL3 facilitates the circularization of circMYO1C, which targets the m6A site of PD-L1 mRNA. By interacting with IGF2BP2, circMYO1C stabilizes PD-L1 mRNA, promoting immune evasion in PDAC^79^. In bladder and breast cancers, METTL3-mediated m6A modification of PD-L1 mRNA stabilizes the mRNA in an IGF2BP1- or IGF2BP3-dependent manner^80^, ^81^. However, in non-small cell lung cancer, METTL3 has been shown to increase the expression of pro-tumorigenic chemokines, including CXCL1, CXCL5, and CCL20, while destabilizing PD-L1 mRNA in an m6A-dependent manner^82^. This suggests that the regulatory role of m6A on immune signaling can vary, and even be opposite, across different cancer types, emphasizing the need to tailor approaches for studying m6A mechanisms based on specific cancer contexts.

**Figure 2:**
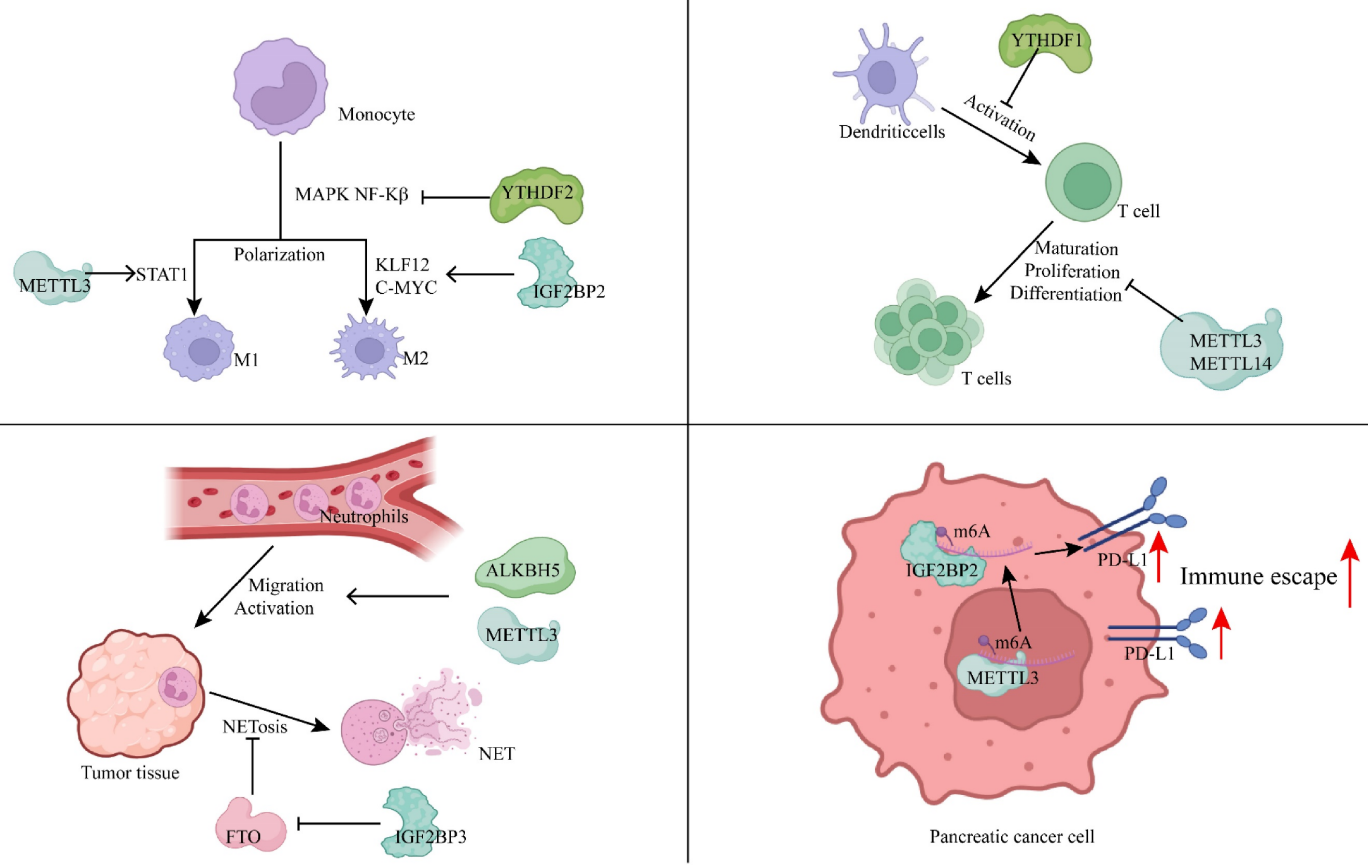
m6A methylation modification modulates the immune microenvironment by regulating immune cells and immune pathways. (Created with BioRender.com).

Immune evasion is a hallmark of tumorigenesis, facilitating tumor proliferation, migration, and invasion. m6A methylation is involved in nearly every aspect of immune evasion, including macrophage development and polarization, T cell proliferation and differentiation, neutrophil migration and activation, and the transduction of immune signaling pathways (Figure 2). Targeting m6A modifications in these processes with therapeutic agents may offer promising strategies for patients who are resistant or have low response rates to conventional chemotherapy.

### 3.4 Tumor resistance mechanisms

Chemotherapy, with GEM as a cornerstone due to its cytotoxic effects on DNA synthesis, is the primary treatment modality for PDAC. GEM-based therapy has remained the first-line treatment for PDAC since its approval in 1997^83^. However, both primary and acquired resistance to chemotherapy have emerged as significant challenges, extending beyond traditional cytotoxic chemotherapy to targeted therapies and immunomodulatory approaches^84^. Understanding the role of m6A methylation modifications in resistance mechanisms holds substantial promise for alleviating this issue and potentially guiding the development of novel therapeutic strategies.

GEM-based combination chemotherapy regimens, often paired with irinotecan, cisplatin, oxaliplatin, and other agents, have provided systemic treatment options with meaningful benefits for patients with advanced or metastatic PDAC^85^. Nevertheless, patients who exhibit resistance or insensitivity to GEM—the cornerstone of these combination therapies—experience significantly reduced clinical benefits. Investigating the mechanistic role of m6A in GEM resistance could provide new insights into overcoming this challenge. The m6A eraser FTO plays a key role in this context by maintaining the stability of long intergenic non-protein coding RNA 1134 (LINC01134) mRNA. FTO reduces the abundance of m6A modifications on LINC01134, preventing RNA degradation after m6A reader YTHDF2 binds to the m6A sites. LINC01134 regulates the WNT signaling pathway through competitive binding to miR-140-3p, promoting PDAC resistance to GEM^86^. In a similar vein, FTO and YTHDF2 cooperatively stabilize the mRNA of Neuronal Precursor Cell-Expressed Developmentally Downregulated 4 (NEDD4), which regulates the PTEN/PI3K/AKT pathway, contributing to GEM resistance in PDAC^87^.

GEMIN5, an m6A mediator identified as a m6A reader, interacts with m6A-modified Fizzy-Related Protein 1 (FZR1) and recruits the eIF3 translation initiation complex, enhancing FZR1 translation. The upregulation of FZR1 maintains the quiescent G0-G1 state in PDAC cells, thereby reducing GEM sensitivity^88^. Another m6A-driven resistance mechanism involves ALKBH5-mediated m6A modification, which promotes the overexpression of the antisense transcript of DNA damage-inducible transcript 4 (DDIT4), encoding the lncRNA DDIT4-AS1. This overexpression enhances PDAC stemness and diminishes GEM chemosensitivity by destabilizing DDIT4 and activating the mTOR pathway^89^. Furthermore, FTO-induced upregulation of KDM5B expression, achieved by stabilizing KDM5B mRNA, contributes to PDAC resistance. KDM5B targets DLG1, which in turn promotes YAP1 nuclear translocation, inducing de novo lipogenesis and contributing to chemotherapy resistance^51^. In PDAC, METTL3 also regulates m6A methylation of the 5′ untranslated region (UTR) of nucleobindin 1 (NUCB1). The m6A reader YTHDF2 binds to these m6A modifications, leading to the downregulation of NUCB1. Notably, NUCB1 plays an essential role in inhibiting PDAC cell proliferation and enhancing GEM efficacy^90^. DNA damage repair represents a major obstacle to the efficacy of chemotherapy in PDAC. Knocking out ZC3H13 downregulates m6A methylation of PHF10, a subunit of the PBAF chromatin remodeling complex. This reduces PHF10 translation in a YTHDF1-dependent manner, inhibiting homologous recombination repair of DNA double-strand breaks^91^.

In a Phase III clinical trial, GEM combined with erlotinib, a human epidermal growth factor receptor type 1 (HER1/EGFR)-targeted agent, demonstrated superior efficacy compared to GEM with placebo, significantly extending overall survival^92^. This highlights the potential of exploring how m6A modifications can augment the sensitivity of targeted therapies or mitigate drug resistance. A study investigating the Nab-paclitaxel and GEM (AG) regimen revealed that Sulindac (K-80003), a drug inhibiting the PI3K/Akt pathway, sensitizes AG through circRNA cFAM124A^93^. Notably, cFAM124A was shown to enhance CTSL expression in an m6A-dependent manner *via* IGF2BP2, resulting in aberrant activation of the PI3K/Akt pathway and increased resistance to GEM. This underscores the importance of investigating whether combining IGF2BP2 inhibitors with current first-line chemotherapy regimens can provide clinical benefits for patients.

m6A also plays a critical role in immunotherapy. Studies have demonstrated that PD-1 checkpoint blockade therapy exhibits diminished efficacy in METTL3-deficient mice, positioning METTL3 as a potential target for tumor immunotherapy^67^. Similarly, improved therapeutic outcomes have been observed with PD-L1 checkpoint blockade in YTHDF1-deficient DCs^70^. In contrast, the loss of YTHDF2 in CD8 T cells accelerates tumor progression and impairs response to PD-1 blockade, both in mice and humans^94^. In PC, PD-1 and PD-L1 expression is closely associated with several m6A regulators, with the strongest correlations found between PD-1 and RBM15, and between PD-L1 and WTAP^78^. Furthermore, METTL3 upregulation in PC elevates lncRNA MALAT1 levels, which in turn enhances PD-L1 expression, potentially enhancing the efficacy of PD-L1-directed immunotherapy.

**Figure 3:**
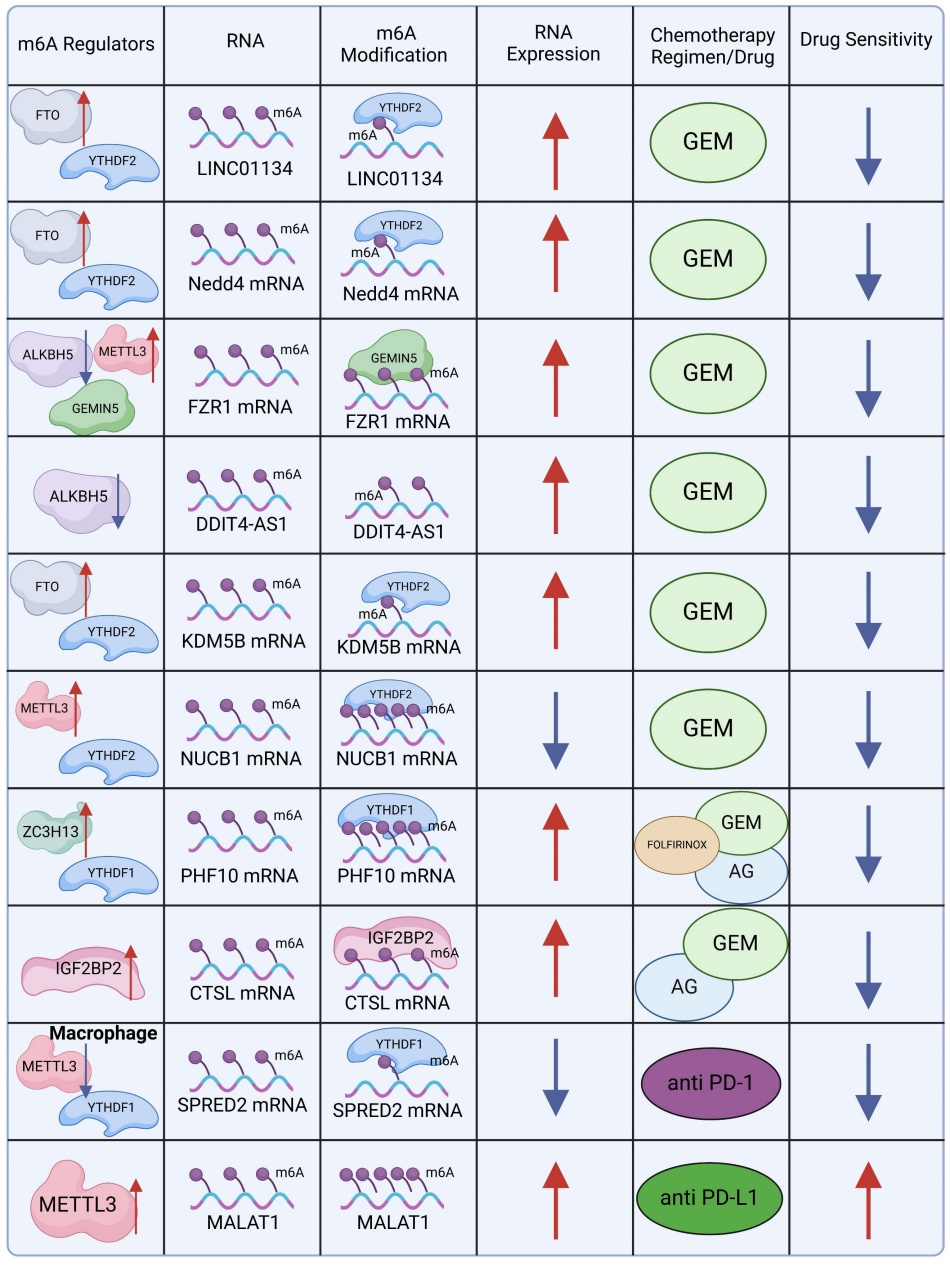
Molecular mechanism of m6A in chemotherapy resistance. (Created with BioRender.com).

The exploration of PDAC resistance mechanisms has progressively revealed the essential role of m6A modifications in mediating resistance to therapies (Figure 3). m6A modifications are implicated across cytotoxic, targeted, and immunomodulatory therapies, suggesting that targeted modulation of m6A could improve the efficacy of existing chemotherapy and extend survival in patients with PDAC.

## 4. The interaction between m6A and ncRNA in PC

Approximately 75% of the human genome is transcribed into RNA, with only 3% transcribed into protein-coding mRNA^95^. ncRNAs, including miRNA, lncRNA, circRNA, and PIWI-interacting RNA (piRNA), perform essential physiological functions in processes such as apoptosis, metastasis, invasion, migration, and cellular proliferation across various cancers^96^. The m6A methylation of ncRNAs plays a critical role in regulating both pro-tumorigenic and tumor-suppressive mechanisms during different stages of cancer progression.

MiRNAs, a subclass of ncRNAs, consist of short RNA molecules, typically 19 to 25 nucleotides in length, that regulate post-transcriptional silencing of target genes. A single miRNA can target hundreds of mRNAs, influencing the expression of numerous functionally interacting genes^97^. MiRNAs are central to carcinogenesis, classified into two categories: tumor-suppressive miRNAs and oncogenic miRNAs. Dysregulation of either category contributes to tumorigenesis^98^. Amphoteric regulatory protein (AREG) serves as an independent prognostic marker in PC, with METTL3 enhancing AREG mRNA stability *via* m6A methylation. In contrast, miR-33a-3p inhibits METTL3, thereby suppressing PC proliferation, migration, and invasion^99^. Additionally, miR-380-3p in PC is enriched with m6A modifications. Silencing METTL3 and METTL14 to remove these modifications synergistically reduces miR-380-3p expression in PC cells, thus inhibiting cell proliferation, migration, and epithelial-mesenchymal transition^100^. While miRNAs regulate m6A modifications, m6A can also directly modify miRNAs to influence their biological functions.

LncRNAs, which are typically longer than 200 nucleotides and can extend beyond 100,000 nucleotides, do not encode proteins^101^. Increasing evidence highlights the critical roles of lncRNAs in various biological processes associated with human diseases. Meta-analyses indicate that lncRNA dysregulation is linked to the overall survival of patients with PC, suggesting their potential as biomarkers for diagnosis and prognosis in PC^102^. As noted earlier, the interaction between m6A modifications and lncRNAs plays a pivotal role in the development and progression of PC. In PC, METTL3 and YTHDF1 promote the upregulation of lncRNA FOXD1-AS1 levels *via* m6A-dependent modifications, enhancing tumorigenesis and the self-renewal of cancer stem cells^103^. Cao *et al.* constructed an m6A-related lncRNA model using TCGA data, analyzing the impact of m6A-related lncRNAs on immune function, including effects on cytolytic activity, inflammation, T cell co-inhibition, checkpoint regulation, and T cell co-stimulation in tumors^104^. The m6A modification of lncRNA ANRIL regulates GEM resistance in PC by modulating the splicing of ANRIL by Serine/arginine-rich splicing factor 3 (SRSF3)^105^. The interplay between m6A methylation and lncRNAs is involved in nearly every process governing PC development, including cell proliferation, migration, immune evasion, and drug resistance.

circRNA, a class of RNA molecules with a circular structure, performs a wide range of functions by acting as molecular sponges to regulate cellular activities. It can bind to proteins, modulate gene expression as a trans-acting factor, and influence RNA methylation levels, including m6A modifications^106^. For instance, circMYO1C targets the m6A site on PD-L1 RNA, enhancing its stability in collaboration with IGF2BP2, thereby facilitating immune evasion in PDAC^79^. Recent studies have further highlighted the role of m6A modifications in circRNA-mediated regulation of innate immunity. Exogenous circRNAs serve as potent adjuvants, promoting antigen-specific T cell activation, antibody production, and anti-tumor immunity *in vivo*. However, m6A modifications suppress immune gene activation and diminish the adjuvant effects of circRNAs^107^. The Ye team developed a bioinformatics tool, Circm6A, which identified 8,807 m6A-circRNAs. In PDAC tumor tissues, m6A-circRNAs exhibit hypermethylation compared to normal tissues. These hypermethylated m6A-circRNAs are implicated in the dysregulation of several critical cancer-related pathways through the formation of circRNA-mRNA co-expression networks^108^.

Moreover, exosomes, which carry a variety of ncRNAs, have emerged as key biomolecules in intercellular communication^109^. The potential of exosomes to deliver ncRNAs to PDAC tissues, thereby influencing cancer progression *via* m6A methylation modifications, presents an intriguing avenue for therapeutic intervention, particularly for patients with advanced PDAC.

## 5. Conclusions and Perspectives

**Figure 4:**
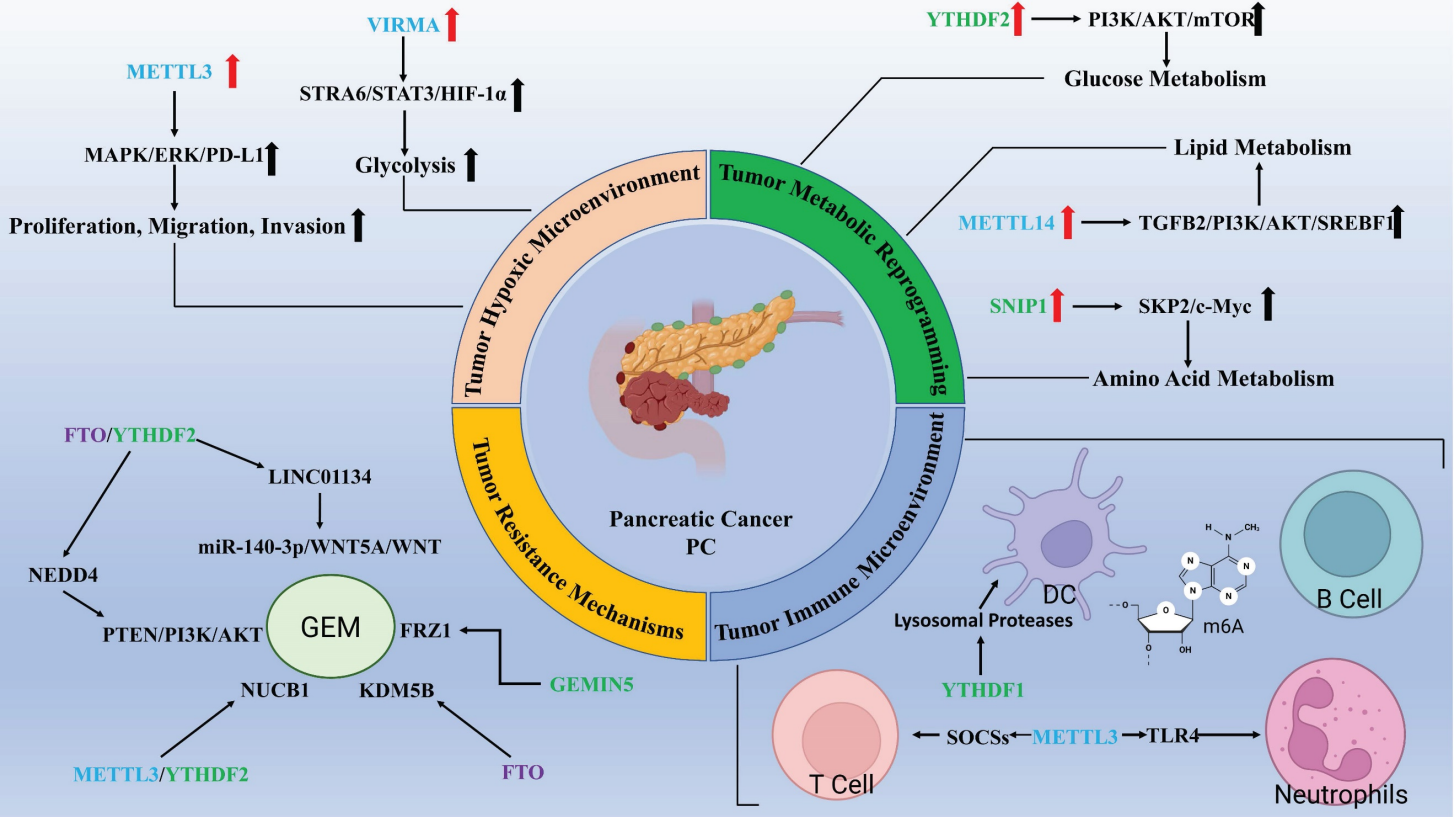
Extensive role of m6A methylation modification in pancreatic cancer. (Created with BioRender.com).

In recent years, epigenetic regulation has garnered increasing attention in cancer research, with RNA m6A modification emerging as a pivotal area of investigation. Numerous studies have highlighted its role in various biological processes of PC, often referred to as the "king of cancers." To underscore the significance of m6A modification in PC, this review explores its involvement in four key aspects of tumor progression: the hypoxic microenvironment, metabolic reprogramming, immune microenvironment, and resistance mechanisms (Fig. 4). m6A modifications are ubiquitous, promoting tumor adaptation to hypoxic conditions while inhibiting immune cell recruitment in such environments. Additionally, m6A modifications participate in metabolic reprogramming in PC, including the well-characterized Warburg effect. Crucially, m6A also regulates the immune microenvironment of PC, influencing immune evasion through interactions with various immune cells, including T cells, B cells, NK cells, DCs, and neutrophils, as well as immune-related pathways such as PD-L1 expression. Furthermore, m6A plays a key role in drug resistance mechanisms, whether in cytotoxic, targeted, or immunomodulatory therapies. This review also introduces the interaction between m6A modifications and ncRNAs. Despite the fact that most ncRNAs do not encode proteins, recent studies have shown that they are essential in regulating cancer cell proliferation, migration, and invasion, interacting with m6A methylation to jointly govern PC progression.

However, it is evident that research on m6A in PC remains in its early stages. Our understanding of the detailed mechanisms behind m6A modification in PC is still limited. This review primarily addresses the regulation of m6A methylation by m6A regulators. In reality, mutations in m6A sites may alter RNA m6A modifications, disrupting post-transcriptional regulation and contributing to tumorigenesis^15^. Moreover, mutations or alterations in m6A regulators can lead to abnormal cellular m6A levels or disrupt biological functions mediated by m6A methylation^16^. The interaction between m6A and other epigenetic factors also plays a significant role in PC progression. Therefore, future research should not be constrained by existing knowledge but should aim for more comprehensive and integrative studies. This will involve incorporating the complexity of m6A modifications into a unified model, identifying novel biomarkers and therapeutic targets, and exploring their potential clinical applications.

In conclusion, m6A-mediated epigenetic regulation stands as a key frontier in PC research. However, the intricate mechanisms of m6A demand systematic, in-depth investigations to elucidate its interactions with the malignant progression of PC, thereby paving the way for more personalized treatment approaches in clinical settings.

## Figures and Tables

**Table 1:** The function of m6A regulators in pancreatic cancer

Type	m6A regulators	Function	Target	Biological Function	Roles in PC	References
Writers	METTL3	Catalyzes m6A modification	TLR4 (neutrophil)	Promote neutrophil activation	—	19,74
	METTL3	Catalyzes m6A modification	SOCS (T cell)	Promote naive T cells homeostasis, proliferation, and differentiation	antitumor	19,69
	METTL3	Catalyzes m6A modification	STAT1 (macrophage)	Promote macrophage M1 polarization	antitumor	19,66
	METTL3	Catalyzes m6A modification	ITGB1/HK2/BCAN-AS1/MALAT1/circMYO1C/FZR1/NUCB1/AREG/miR-380-3p/FOXD1-AS1	Promote proliferation, metastasis, invasion, immune escape, glycolysis, drug resistance, and PNI	oncogenic	19,27,30,56,78,79,88,90,99,100,103
	METTL5	Promotes the m6A methylation of 18 S rRNA	—	—	—	16
	METTL14	Forms heterodimer with METTL3 to catalyze m6A modification	TGFB2/circSTX6/miR-380-3p	Promote proliferation, metastasis, and drug resistance	oncogenic	19,50,53,100
	METTL16	Catalyzes m6A modification	—	—	—	19
	WTAP	Regulatory subunit of m6A methyltransferase and recruits METTL3 and METTL14 into the nuclear speckles	GBE1	Promote proliferation	oncogenic	19,49
	RBM15	Directs METTL3-METTL14 heterodimer to specific RNA sites	—	Inhibit macrophage infiltration and phagocytosis by macrophages	oncogenic	19,63
	RBM15B	Directs METTL3-METTL14 heterodimer to specific RNA sites	—	—	—	19
	VIRMA/KIAA1429	Recruits METTL3 and METTL14, and direct m6A methylation at specific sites to induce mRNA splicing and RNA processing.	STRA6	Promote metastasis	oncogenic	19,38
	PCIF1	Catalyzes 5′ m6Am* methylation of capped mRNAs	—	—	—	20
	ZC3H4	Mediates the m6A methylation of U2 snRNA to regulate pre-mRNA splicing	—	—	—	16
	ZC3H13	Bridges WTAP to the mRNA-binding factor Nito; Anchors WTAP in the nucleus to enhance m6A modification	PHF10	Promote drug resistance	oncogenic	16,91
Erasers	FTO	Acts as m6A demethylase to promote mRNA splicing and translation	SNAI1/KDM5B/LINC01134/NEDD4	Promote proliferation, drug resistance, and EMT	oncogenic	19,32,51,86,87
	ALKBH5	Removes m6A modification to promote mRNA nuclear processing and mRNA export	CXCR2/NLRP12/PTGER4 (neutrophil)	Promote neutrophil migration	—	19,73
	ALKBH5	Removes m6A modification to promote mRNA nuclear processing and mRNA export	FZR1	Inhibit drug resistance	antitumor	19,88
	ALKBH5	Removes m6A modification to promote mRNA nuclear processing and mRNA export	HDAC4/FABP5/DDIT4-AS1	Promote proliferation, metastasis, glycolysis, drug resistance and lipid metabolism	oncogenic	19,45,52,89
Readers	YTHDC1	Promotes mRNA splicing and transcriptional silencing	—	—	—	19
	YTHDC2	Improves the translation efficiency of target mRNA	—	—	—	19
	HNRNPA2B1	Promotes primary miRNA processing and mRNA splicing	—	—	—	19
	HNRNPC11	Mediates mRNA splicing and maturity	—	—	—	16
	HNRNPG	Mediates mRNA splicing and maturity	—	—	—	16
	YTHDF1	Promotes mRNA translation initiation	transcripts encoding lysosomal proteases (DC)	Inhibit the cross-presentation of tumor antigens and the cross-priming of CD8+ T cells	oncogenic	19,70
	YTHDF1	Promotes mRNA translation initiation	PHF10/FOXD1-AS1	Promote drug resistance	oncogenic	19,91,103
	YTHDF2	Promotes mRNA degradation	MAP2K4/MAP4K4/STAT1/PPAR- γ (macrophage)	Inhibit macrophage polarization and proinflammatory cytokine secretion	oncogenic	19,65
	YTHDF2	Promotes mRNA degradation	PTEN/NEDD4/NUCB1	Promote proliferation, migration, invasion, drug resistance, and the Warburg effect	oncogenic	19,48,87,90
	YTHDF2	Promotes mRNA degradation	HDAC4/KDM5B/LINC01134	Inhibit migration, glycolysis, and drug resistance	antitumor	19,45,51,86
	YTHDF3	Interacts with YTHDF1 to promote mRNA translation or interacts with YTHDF2 to promote mRNA degradation	DICER1-AS1	Promote proliferation and glycolysis	oncogenic	19,47
	IGF2BP1	Promotes the stability and translation of mRNA	—	—	—	19
	IGF2BP2	Promotes the stability and translation of mRNA	STRA6/TGFB2/PACERR/KLF12/C-Myc/PD-L1/CTSL	Promote proliferation, migration, invasion, glycolysis, and drug resistance	oncogenic	19,38,50,64,79,93
	IGF2BP3	Promotes the stability and translation of mRNA	MIB1 (neutrophil)	Promote NET	—	19,77
	IGF2BP3	Promotes the stability and translation of mRNA	GBE1	Promote proliferation and stemness-like properties	oncogenic	19,49
	GEMIN5	Recruiting eIF3 complex to promote mRNA translation	FZR1	Promote drug resistance	oncogenic	88
	KHSRP	Promotes the stability and translation of mRNA	FAK	Promote proliferation, migration, and invasion	oncogenic	25
	eIF3	Promotes mRNA translation	—	—	—	19
	FMRP	Promotes the nuclear export and stability of m6A-modified RNAs	—	—	—	16
	PRRC2A	Binds to a consensus GGACU motif in the Olig2 coding sequence to stabilize Olig2 mRNA	—	—	—	16
	RBM33	Forms a complex with ALKBH5 and mediates m6 A demethylation of selected transcripts by regulating ALKBH5 substrate accessibility and activity	—	—	—	16

m6Am: N6,2′-O-dimethyladenosine

## Abbreviations

SAM: S-adenosyl methionine
HIF: Hypoxia Inducible Factor
MAPK: Mitogen-activated protein kinase
CAFs: Cancer-associated fibroblasts
ERK: Extracellular signal-regulated kinase
PTEN: Phosphatase and tensin homolog deleted on chromosome ten
PD-L1: Programmed cell death-Ligand 1
PI3K: Phosphatidylinositol 3-kinase
ITGB1: Integrin Beta 1
mTOR: Mammalian target of rapamycin
GBE1: Glycogen branching enzyme 1
SMAD: Mothers against decapentaplegic homolog
DLG1: Discs Large Homolog 1
YAP1: Yes-Associated Protein 1
KDM5B: Lysine-specific demethylase 5B
AA: Amino acid
SNIP1: SMAD nuclear-interacting protein 1
SKP2: S phase kinase-associated protein 2
KLF12: Kruppel-like factor 12
NF-κB: Nuclear factor-kappa B
PPAR-γ: Peroxisome proliferator-activated receptor γ
STAT1: Signal transducer and activator of transcription 1
SOCS: Suppressor of cytokine signaling
CXCR2: CXC-Chemokine Receptor 2
NLRP12: NOD-like receptors family pyrin domain containing 12
PTGER4: Prostaglandin E Receptor 4
TNC: Tenascin C
WNK1: WNK lysine deficient protein kinase 1
NETs: Neutrophil extracellular traps
CXCL1: CXC-chemokine ligand 1
CCL20: C-C chemokine ligand 20
NUCB1: Nucleobindin-1
NEDD4: Neuronal precursor cell-expressed developmentally downregulated 4
FABP5: Fatty Acid Binding Protein 5
